# Overexpression of sema3a in myocardial infarction border zone decreases vulnerability of ventricular tachycardia post-myocardial infarction in rats

**DOI:** 10.1111/jcmm.12035

**Published:** 2013-05-27

**Authors:** Ren-Hua Chen, Yi-Gang Li, Kun-Li Jiao, Peng-Pai Zhang, Yu Sun, Li-Ping Zhang, Xiang-Fei Fong, Wei Li, Yi Yu

**Affiliations:** a Department of Cardiology, School of Medicine, Xinhua Hospital, Shanghai Jiaotong UniversityShanghai, China; b Department of Cardiology, Ganzhou People Hospital, Ganzhou Hospital Affiliated to Nanchang UniversityGanzhou, Jiangxi, China; c Department of Ultrasound, School of Medicine, Xinhua Hospital, Shanghai Jiao Tong UniversityShanghai, China

**Keywords:** myocardial infarction, Sema3a, reinnervation, ventricular tachyarrhythmia

## Abstract

The expression of the chemorepellent Sema3a is inversely related to sympathetic innervation. We investigated whether overexpression of Sema3a in the myocardial infarction (MI) border zone could attenuate sympathetic hyper-innervation and decrease the vulnerability to malignant ventricular tachyarrhythmia (VT) in rats. Survived MI rats were randomized to phosphate buffered saline (PBS, *n* = 12); mock lentivirus (MLV, *n* = 13) and lentivirus-mediated overexpression of Sema3a (SLV, *n* = 13) groups. Sham-operated rats served as control group (CON, *n* = 20). Cardiac function and electrophysiological study (PES) were performed at 1 week later. Blood and tissue samples were collected for histological analysis, epinephrine (EPI), growth-associated factor 43 (GAP43) and tyrosine hydroxylase (TH) measurements. QTc intervals were significantly shorter in SLV group than in PBS and MLV groups (168.6 ± 7.8 *vs*. 178.1 ± 9.5 and 180.9 ± 8.2 ms, all *P* < 0.01). Inducibility of VT by PES was significantly lower in the SLV group [30.8% (4/13)] than in PBS [66.7% (8/12)] and MLV [61.5% (8/13)] groups (*P* < 0.05). mRNA and protein expressions of Sema3a were significantly higher and the protein expression of GAP43 and TH was significantly lower at 7 days after transduction in SLV group compared with PBS, MLV and CON groups. Myocardial EPI in the border zone was also significantly lower in SLV group than in PBS and MLV group (8.73 ± 1.30 *vs*. 11.94 ± 1.71 and 12.24 ± 1.54 μg/g protein, *P* < 0.001). Overexpression of Sema3a in MI border zone could reduce the inducibility of ventricular arrhythmias by reducing sympathetic hyper-reinnervation after infarction.

## Introduction

Sudden cardiac death (SCD) remains a major and unresolved public health problem. Previous myocardial infarction (MI) was identified in 75% of SCD victims 1, 2. In most cases, the direct cause of SCD is ventricular fibrillation (VF), which is usually preceded by ventricular tachycardia (VT). Life-threatening ventricular arrhythmia is thus an important cause of mortality post-MI 3, 4. Previous studies demonstrated that sympathetic hyper-innervation was associated with arrhythmogenesis and SCD and augmented sympathetic nerve regeneration could increase the incidence of VT, VF and SCD in chronic MI animal models 5–10.

Semaphorins are a family of secreted and membrane-anchored glycoproteins 11 and are important in repulsive axon guidance during neuroembryogenesis 12. Sema3a, one of the best characterized members in this family, is a diffusible molecule which could induce growth cone collapse and axon repulsion of several neuronal populations 13–16. Down-regulated expression of Sema3a mRNA in adult neurons was observed in post-peripheral nerve injury 17, 18 and down-regulation of semaphorin expression could result in a more permissive environment for axonal regeneration and sprouting.

In transgenic mice, Sema3a expression was inversely related to sympathetic innervation, cardiac-specific Sema3a overexpression reduced sympathetic innervation, however, increased susceptibility to ventricular arrhythmias 19. It remains unknown now whether local overexpression Sema3a in the MI border zone may decrease or increase the susceptibility to ventricular arrhythmias. We observed the effect of Sema3a overexpression in the MI border zone on the susceptibility to ventricular arrhythmias in a rat MI model.

## Materials and methods

### Lentivirus production

A third generation of self-inactivating lentivirus vector was purchased from Shanghai Innovation Biotechnology Co. Ltd (Shanghai, China), which contains a cytomegalovirus -driven green-fluorescent protein (GFP) reporter gene and an EF1-alpha promoter upstream of cloning restriction sites (ClaI and mluI) to allow introduction of oligonucleotides encoding short hairpin RNAs (shRNAs). The sequence of the siRNA targeting rat Sema3a, 5′-GGATGGGTCCTCATGCTCAC-3′, has previously been proven to efficiently down-regulate rat Sema3a 20. A missense siRNA, with the sense sequence 5′-CGAGCAGAAAGGATTGAAA-3′ which lacks complementary sequences in the murine genome, was used as a control. A BLAST search was carried out to avoid unintentional silencing of non-target host cell genes (http://www.ncbi.nlm.nih.gov). Oligos were chemically synthesized, annealed and cloned into the shRNA lentivector between the ClaI and mluI sites of the plasmid. Correct insertions of shRNA cassettes were confirmed by direct DNA sequencing. GeneBank accession number is 017310. Recombinant lentivirus was produced by cotransfecting 293T cells with the lentivirus expression plasmid and packaging plasmids using calcium phosphate method 21. Infectious lentivirus was harvested at 72 hrs after transfection, centrifuged to eliminate cell debris and then filtered through 0.22 μm cellulose acetate filters. Infectious titre was determined by fluorescence-activated cell sorting analysis of GFP positive in 293T cells. Virus titres were in the range of 10^8^ transducing units/ml medium.

### MI and Lentiviral vector microinjection

Sixty male adult Spraque–Dawley rats were randomized to three groups and subjected to MI operation (*n* = 20 each): (i) phosphate buffered saline (PBS); (ii) mock lentivirus (MLV) and (iii) lentivirus-mediated overexpression of Sema3a (SLV). MI was induced by ligation of the left anterior descending (LAD) coronary artery as previously described 22. Ligation was deemed successful when the anterior wall of the left ventricle turned pale. Six hours post-MI, six intramyocardial PBS, MLV and SLV injections at MI border zone (50 μl each) were performed under direct observation using a 31-gauge needle in rats with echocardiographic determined left ventricular ejection fraction (EF) <50%. Reporter gene expression was verified by fluorescence microscopy after 1 week for identifying transducted lentiviral vector in the recipient heart. The observation duration was set at 1 week post-MI because sympathetic reinnervation has been shown to be present at 6 days after injury 23. Sham-operated rats served as normal control group (CON, *n* = 20). The animal experiment was approved by the medical ethics committee of Xinhua Hospital, Shanghai Jiaotong University, School of Medicine, and conforms to the Principles of Laboratory Animal Care (National Society for Medical Research) and the Guide for the Care and Use of Laboratory Animals (NIH). All evaluations were carried out in a blinded manner.

### Assessment of cardiac function by echocardiography

Left ventricular (LV) ejection fraction (EF) was measured with M-mode (Vevo 770 System, and 15-MHz probe, VisualSonics Inc, Toronto, ON, Canada) at baseline, at 5 hrs and at 1 week post-LAD ligation by single operator blinded to the experimental designs in sedated rats with pentobarbital (40 mg/kg i.p; Pentobarbital sodium, Fluka, Sweden).

### Ventricular PES in sedated rats

After echocardiographic examination, all rats underwent programmed electrical stimulation (PES) study by an operator blinded to the experimental designs. Surface six-lead ECGs were recorded using 25-gauge subcutaneous electrodes connected to a recording system through an analogue-digital converter for monitoring and off-line analyses. ECG channels were filtered out below 10 Hz and above 100 Hz. Standard criteria were used for interval measurements: RR, PR, QRS and QT, and QTc (QT interval corrected for the heart rate using Bazett's formula). A 1.9 F eight-polar catheter for small animal electrophysiology (Scisence, Ontario, Canada) was advanced from the right external jugular vein to the right atrium and through the tricuspid valve to the right ventricle. Pacing was performed by applying a 1-ms pulse pacing of width at two times higher than capture threshold. Standard clinical PES protocols were used, including burst, single, double and triple extra stimuli applied following a train of nine stimuli at 100-ms drive cycle length. The coupling interval of the last extra stimulus was decreased by 2-ms steps from 80 ms down to the ventricular effective refractory period (VERP). PES protocols were interrupted if sustained VT or VF was induced. Sustained VT was defined as fast ventricular rhythm of 15 or more beats, according to Lambeth Conventions 24. No discrimination was made between monomorphic and polymorphic VT. Arrhythmia scoring system established by Curtis *et al*. was used (Table 1) 25.

**Table 1:** Arrhythmia scoring system

	Salvo	BVT	SVT	NVT	VF
S_1_S_1_∼S_1_S_2_S_3_	0	1	1.5	2	2.5
Burst stimuli	0	0.5	1	1.5	2.0

S_1_S_1_∼S_1_S_2_S_3_: single, double and triple extra stimuli; Salvo: two or three consecutive ventricular premature beats; BVT: burst ventricular tachycardia, more than three, but less than 15 ventricular premature beats; SVT: ventricular tachycardia reverted to sinus rhythm spontaneously; NVT: ventricular tachycardia did not reverted to sinus rhythm spontaneously and required thump-version; VF: ventricular fibrillation.

### Tissue preparation

Post-PES all rats were killed in deep anaesthesia with overdose pentobarbital (80 mg/kg bodyweight) and hearts were excised and rinsed in cold PBS. For histological analysis, hearts were perfuse fixed with 4% paraformaldehyde, and frozen at 4°C. For cryosection, fresh tissues were snap frozen in liquid nitrogen, embedded in OCT compound in cryomoulds and stored at −80°C. For Western blot analysis and ELISA, fresh MI border zone tissues were stored in liquid nitrogen and then proceed to standard tissue protein extraction procedure.

### ELISA for epinephrine (EPI) levels

Blood samples of rats were collected before PES and immediately centrifuged at 3,000 × *g* for 10 min., and the serum was stored at −80°C until analysis. EPI levels of serum and MI border zone tissue protein extraction were measured using a commercial ELISA kit (R&D, Minneapolis, MN, USA).

### Real-time PCR analysis

The analyses of Sema3a mRNA were performed as follows: Total RNA was extracted from the samples using TRIzol (Invitrogen, Carlsbad, CA, USA). cDNA was synthesized by the Toyobo First-Strand Synthesis kit (TOYOBO, Osaka, Japan) according to the manufacturer's protocols. PCR amplification was carried on Gene Rotor 3000 using TOYOBO real-time PCR kit. Primers' (*GAPDH-F:TTCAACGGCACAGTCAAGG; GAPDH-R:CTCAGCACCAGCATCACC*; *Sema3a-F:*AGACGACAGGATATAAGGAATGG; *Sema3a-R:GCAGACTACGAAGCAGGAG*) concentration was 200 pM. PCR conditions were: denaturation (95°C, 1 min.); 40 cycles of denaturation (95°C, 15 sec.) and annealing (60°C, 15 sec.). GAPDH was used as internal reference transcript. Relative quantification of Sema3a was performed by modified real-time RT-PCR method. Briefly, total RNAs were extracted from the samples using TRIzol (Invitrogen). Five microgram total RNA was polyadenylated by Poly(A) Tailing Kit (Ambion, St. Austin, TX, USA), and oligo (dT) primers were used to reversely transcribe the poly(A)-tailed miRNAs into cDNAs. Then miRNA was amplified by SYBR Green real-time PCR using the miRNA-specific forward primer. PCR amplification was carried on Gene Rotor 3000 using real-time PCR kit (TOYOBO). Primers' (*GAPDH-F:TTCAACGGCACAGTCAAGG*; *GAPDH-R:CTCAGCACCAGCATCACC*; *Sema3a-F:*AGACGACAGGATATAAGGAATGG; *Sema3a-R:GCAGACTACGAAGCAGGAG*) concentration was 200 pM. PCR conditions were as follows: denaturation (95°C, 1 min.); 40 cycles of denaturation (95°C, 15 sec.), annealing (60°C, 15 sec.). The quantity of miRNA, relative to a reference gene, was calculated using the formula, 2^−ΔCT^, where ΔCT = (CT_miRNA_ − CT_reference RNA_). The comparison of miRNA expression was based on a comparative CT method (ΔΔCT), and the relative miRNA expression was quantified according to the formula of 2^−ΔΔCT^, where ΔΔCT = (CT_miRNA_ − CT_reference RNA_) − (CT_calibrator_ − CT_reference RNA_). GAPDH was used as internal reference transcript.

### Western blot analysis

Western blot analysis was performed with 8% (for Sema3a) or 12% (for GAP43 and Tyrosine Hydroxylase, TH) polyacrylamide gels. Each lane was loaded with an equal amount of protein extracts, transferred to PVDF membrane for 1 hr (for GAP43 and TH) or 2 hrs (for Sema3a). Blots were stained with ponceau to verify equal loading, and transfer of proteins membranes was blocked and probed overnight at 4°C with primary antibodies. Membranes were then incubated with horseradish peroxidase conjugated secondary antibodies for 2 hrs at room temperature. The information of antibodies was showed in Table 2. The intensity of the signal was determined using the ECL-Plus detection system and Bio-Rad imaging system. Imagines were adjusted by glyceraldehyde phosphate dehydrogenase (GAPDH) and analysed by Quantity One software (Bio-Rad, Hercules, CA, USA).

**Table 2:** Primary and secondary antibodies for Western blot analysis used

	Company	Dilution
Primary antibody		
Rabbit anti-human Sema3A antibody	Santa Cruz	1:200
Rabbit anti-rat Tyrosine Hydroxylase antibody	Enzo Life Sciences	1:500
Rabbit anti-rat Growth-associated Factor 43 antibody	Abcom	1:5000
Secondary antibody		
Goat anti-rabbit HRP conjugated	Jackson Immuno-Research	1:1000

### Histological analysis

Five micrometre sections were cut, stained with Masson's trichrome and mounted. The inner and outer perimeters of the LV were traced with a digital image processing system (Leica Qwin V3, Wetcules, Germany). Infarct size (%) was expressed as the mean of the percentage of infarcted LV *versus* total LV inner and outer circumferences 26.

### Immunostaining for visualization of sympathetic innervation

An indirect immunofluorescence technique was adopted for the visualization of sympathetic innervation. Cryostat sections (5 μm thick) were cut and mounted on silica gel coated slides, stored at −80°C until use. Before staining, slides were warmed at room temperature for 1 hr and fixed in ice-cold acetone for 10 min., air dried for 1 hr and washed in PBS and proceeded to standard staining procedure. Cryostat sections stained with antibodies to β-actinin (Santa Cruz, Dallas, TX, USA), GAP43 (a marker peptide for neuronal regeneration and outgrowth, Abcom Ltd, Hongkong) and TH (Enzo Life Sciences, Farmingdale, NY, USA) to detect cardiomyocytes and sympathetic nerve fibres respectively. The sections were incubated with secondary antibodies conjugated with Rhodamine (Jackson Immuno Research, West Grove, PA, USA) and the nuclei were stained with 4′,6-diamidino-2-phenylindole (DAPI) and examined with a Leica microscope equipped for epillumination. In negative control experiments, no immunofluorescence staining was obtained when sections were incubated without the primary antibody, with pre-immune serum as replacement for the primary antibody. The procedure was performed by an investigator blinded to the protocol.

### Data analysis

Data are presented as mean ± SD and compared by one-way anova followed by Turkey's *post hoc* test for individual significant difference. Electrophysiological data (scoring of programmed electrical stimulation-induced VT/VF) were compared with Chi-squared test. The significant level was assumed at a value of *P* < 0.05.

## Results

### Mortality post-LAD and microinjection

Mortality post-LAD ligation and microinjection was similar among three groups (20% in the PBS, 25% in the MLV and 30% in the SLV group, *P* > 0.05). Mortality after sham operation was zero.

### Infarct size, LVEF post-MI and microinjection

**Fig. 1:**
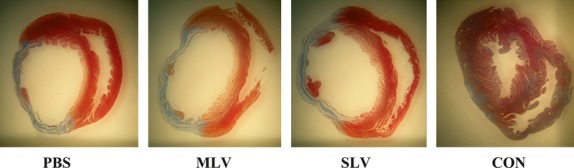
Masson's trichome, blue indicates infarct scar (*n* = 12 in PBS group, *n* = 13 in MLV group and *n* = 13 in SLV group, *n* = 20 in CON group).

**Fig. 2:**
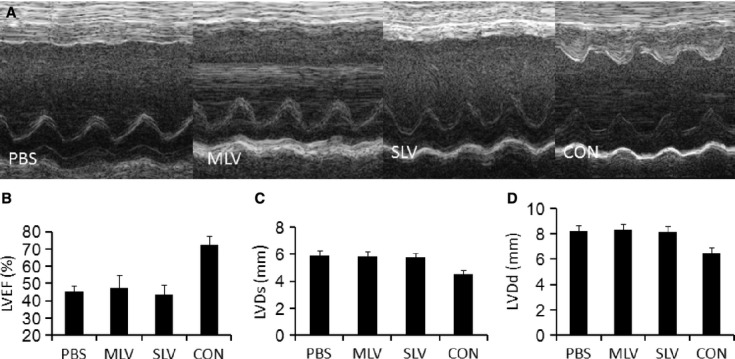
Serial analysis of 2D mode echocardiography of CON, PBS, MLV and SLV groups (**A**). LVEF in the three MI groups was similar at 1 week after infarction and microinjection, *P* > 0.05 (**B**), also the LVDs and LVDd (**C** and **D**).

One week after infarction and microinjection, the infarct region of the LV was very thin and partly replaced by scar tissue, which was not affected by injection of PBS, MLV or SLV. Infract size of the SLV, PBS and MLV groups was similar (33.3 ± 2.6%, 31.2 ± 3.5%, 33.8 ± 4.6%, respectively, *P* > 0.05, Fig. 1). Pre-LAD, LVEF was about 75% and similar among all groups. Animals with LVEF ≥ 50% at 5 hrs post-LAD were excluded [MLV(2/15), SLV(1/14) and PBS(4/16)]. LVEF was equally reduced among the three MI groups at 1-week after operation (Fig. 2).

### QTc interval changes and ventricular vulnerability to VT

**Fig. 3:**
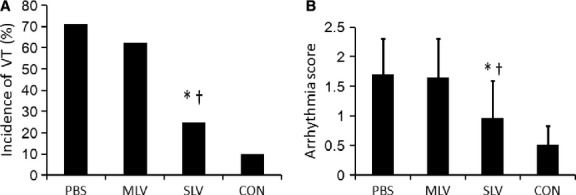
Incidence of VT and arrhythmia score at 1 week after infarction and microinjection. (**A**) Incidence of non–self-terminating VTs during PES. (**B**) Arrhythmia score in the SLV group was significantly lower than that in MLV and PBS groups at 1 week after infarction and microinjecttion. *P = 0.018, *versus* the PBS group and †*P* = 0.025, *versus* the MLV group respectively.

The ECG measurements before electrophysiological test of all rats were summarized in Table 3. The QTc interval was significantly shortened in the SLV group compared with the PBS and MLV groups at 1-week post-LAD and microinjection. The incidence of VT by S_1_S_1_ stimulus at room temperature during PES was significantly lower in SLV group than in PBS and MLV groups (Fig. 3a). Arrhythmia score was also significantly lower in SLV group than in PBS and MLV groups (Fig. 3b). QTc interval, inducibility of VT and arrhythmias score are significantly higher in PBS and MLV groups than in control group and these parameters are similar between control group and SLV group.

**Table 3:** ECG parameters at 1 week after infarction and microinjection

	RR(ms)	P(ms)	PR(ms)	QRS(ms)	QTc(ms)
PBS	168.5 ± 10.0	16.0 ± 1.2	54.9 ± 2.5	23.3 ± 1.5	178.1 ± 9.5
MLV	170.0 ± 7.2	16.2 ± 0.7	55.0 ± 2.5	25.5 ± 1.6	180.9 ± 8.2
SLV	166.0 ± 7.2	16.4 ± 1.6	53.4 ± 3.7	21.0 ± 1.2	168.6 ± 7.8*^†^
CON	167.1 ± 5.3	16.1 ± 1.6	52.5 ± 4.1	22.3 ± 1.7	165.7 ± 8.3

### mRNA and protein expression of Sema3a in MI border zone

**Fig. 4:**
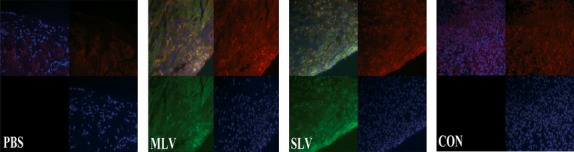
Determination of lentivirus transduction and expression in myocardial infarction border zone at 1 week. GFP expression was observed under fluorescence microscopy. (Blue = nuclei, Green = GFP, Red = β-actin, all ×200).

**Fig. 5:**
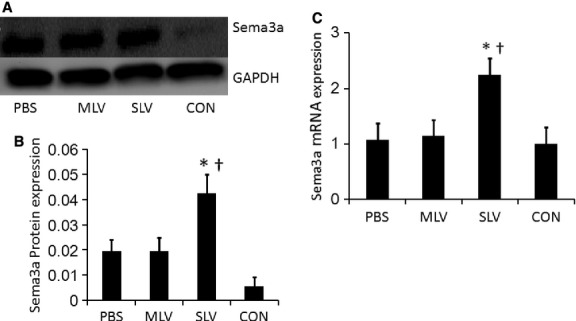
Effect of SLV on Sema3a mRNA and protein expressions. The SLV could significantly up-regulate the Sema3a mRNA and protein level. A representative Western blot (**A**). Relative densitometric values of Western blots (**B**). Values were calculated from three independent experiments; *P < 0.05 *versus* the PBS group, †*P* < 0.05 *versus* the MLV group. (**C**) mRNA expression of Sema3a by RT-PCR in different groups. The values had been normalized to GAPDH measurement and then expressed as a ratio of normalized values to mRNA in static control group; *P < 0.05 *versus* the PBS group, †*P* < 0.05 *versus* the MLV group.

Reporter gene expression examined by fluorescent microscopy demonstrated lentivirus-derived GFP^+^ labelled host cardiomyocytes at 1 week after either MLV or SLV injection, however, GFP^+^ cardiomyocytes cells were not observed in PBS group and in sham group (Fig. 4). The mRNA-Sema3a level in the myocardium of MI border zone in SLV groups was significantly higher (about twofold) at 1 week after transfection compared with PBS, MLV, CON groups (Fig. 5c).

Sema3a protein expression in LV-free wall of sham group was very low and the Sema3a protein expression in the MI border zone from SLV group was significantly higher than that in PBS and MLV group (Fig. 5a and b).

### Nerve sprouting and GAP43 protein expression in MI border zone

**Fig. 6:**
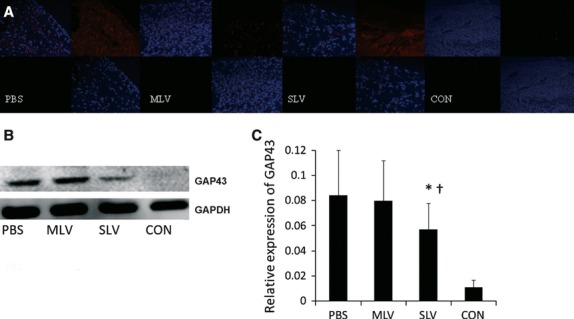
GAP43-positive (GAP^+^) nerves innervation in the myocardial infarction border zone at 1 week after infarction or sham operation. (**A**) The PBS and MLV groups were abundantly innervated by GAP43-positive (red) nerves. (Blue = nuclei, Red = GAP43, all ×200). (**B**) A representative Western blot. (**C**) Relative densitometric values of Western blots. Values expressed as mean ± SD; calculated from three independent experiments; *P < 0.05 *versus* the PBS group, †*P* < 0.05 *versus* the MLV group.

Immunofluorescence staining demonstrated GAP43^+^ nerve fibres in the MI border zone in three MI groups at 1 week after infarction, and GAP43^+^ nerve fibres were more abundant in the PBS and MLV groups than in the SLV group (Fig. 6a). Protein expression of GAP43 in MI border zone was significantly higher than that in LV-free wall of sham group, and protein expression of GAP43 in MI border zone was significantly down-regulated in SLV group compared with that in PBS group and MLV group (Fig. 6b and c).

### Sympathetic innervation and protein expression of TH at MI border zone

**Fig. 7:**
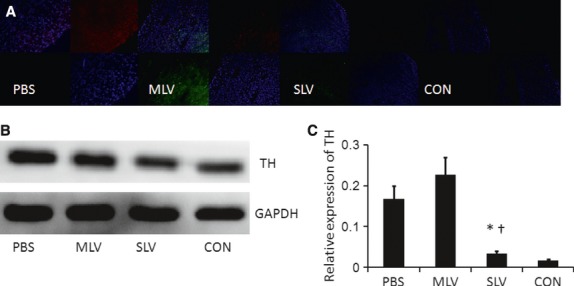
Cardiac sympathetic innervation patterning in the myocardial infarction border zone at 1 week after infarction or sham operation. (**A**) Triple immunostaining for nuclei, tyrosine hydroxylase and GFP in three groups. Tyrosine hydroxylase expression was observed under fluorescence microscopy. (Blue = nuclei, Green = GFP, Red = tyrosine hydroxylase, all ×200). (**B**) Representative Western blot. (**C**) Relative densitometric values of Western blots. Values expressed as mean ± SD, calculated from three independent experiments; *P < 0.05 *versus* the PBS group; †*P* < 0.05 *versus* the MLV group.

The positive TH staining pattern and TH protein expression pattern were similar as changes in GAP43 shown above (Fig. 7).

### Myocardial EPI level in MI border zone

**Fig. 8:**
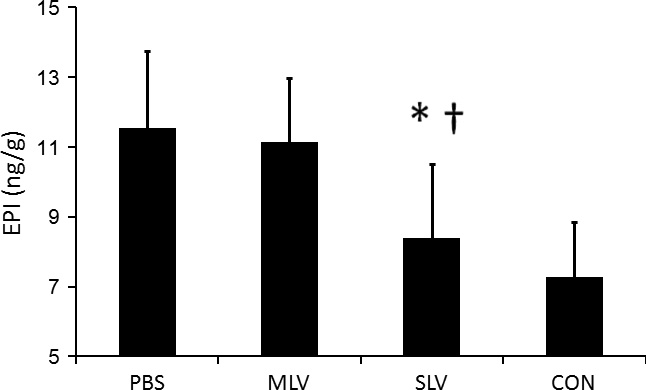
Cardiac epinephrine (EPI) levels assessed by ELISA. EPI was significantly down-regulated in the SLV group than that in the PBS and MLV groups at 1 week after injection. *P < 0.001, *versus* the PBS group; † *P* < 0.001, *versus* the MLV group respectively. (*n* = 13 in PBS group, *n* = 13 in MLV group, *n* = 12 in SLV group and *n* = 20 in CON group).

Although cardiac sympathetic reinnervation was demonstrated by immunofluorescence staining of TH and GAP43, it did not imply that the nerves were also functional. Thus, we determined the circulating and myocardial EPI levels at MI border zone to investigate cardiac sympathetic function. Circulating EPI levels were similar among three MI groups (data not shown). The border zone myocardial EPI levels were significantly down-regulated in SLV group compared with PBS or MLV group (Fig. 8).

## Discussion

In this study, we observed the effect of myocardial overexpression of sema3a on after infarction sympathetic reinnervation and the inducibility of VT by PES in a rat MI model. The major findings were as follow: (i) Local microinjection of SLV-enhanced protein expression of Sema3a in MI border zone; (ii) Myocardial overexpression of Sema3a-attenuated sympathetic reinnervation in MI border zone and (iii) Myocardial overexpression of Sema3a significantly reduced the incidence of PES-induced malignant arrhythmia in this rat MI model.

It is known that expression of Sema3A mRNA in adult neurons is down-regulated after peripheral nerve injury 17, 27. Down-regulation of semaphorin expression could result in a more permissive environment for axonal regeneration and sprouting. This study tested the hypothesis that up-regulation of semaphorin expression might attenuate sympathetic hyper-reinnervation post-MI. Our results showed that microinjection of SLV enhanced mRNA and protein expression of Sema3a in the MI border zone and significantly inhibited nerve sprouting and sympathetic hyper-innervation, decreased prolonged QTc intervals and the inducibility of ventricular tachyarrhythmias during EPS in this rat MI model. Our finding suggested that myocardial overexpression of Sema3a post-MI might be beneficial in terms of reducing malignant arrhythmias related to increased post-injury sympathetic nerve density, which is obviously associated with the occurrence of ventricular arrhythmia and SCD in animal models or MI patients 10, 28, 29. To our best knowledge, this is the first report on the impact of myocardial Sema3a overexpression in this MI model. Consistent with the notion that the nerve sprouting was augmented at the MI border zone 30–32, our results detected excessive sympathetic reinnervation after infarction at the MI border zone, and this process could be inhibited by myocardial Sema3a overexpression in this MI model.

Previous reports showed that MI could induce significant nerve sprouting and sympathetic hyper-innervation, prolonged APD and QTc intervals and increased repolarization dispersion 33. Cardiac nerve injury caused by MI could also trigger the reexpression of Sema3a and other neurotrophic factor genes in the non-neural cells around the site of injury 34–36, leading to nerve regeneration through nerve sprouting, 9, 37, 38 whereas disturbance on Sema3a expression system was associated with compensatory sprouting and synaptic remodelling 39. Consistent with above findings observed in the peripheral nervous system, we showed that Sema3a overexpression in rat heart could also inhibit cardiac nerve sprouting and attenuate sympathetic hyper-innervation post-MI in a rat model. The impact of myocardial overexpression on different animal models seemed not to be identical. In a mice transgenic model, overexpression of Sema3a in the heart resulted in both reduced sympathetic innervation and increased susceptibility to ventricular arrhythmias as a result of catecholamine supersensitivity and prolongation of the action potential duration 21. The underlying mechanisms for the divergent results observed in this rat MI model and the transgenic mice model remains unknown now. It is possible that the myocardial Sema3a overexpression in a post-MI model might help to restore the myocardial Sema3a level to the pre-MI status, thus, be beneficial on inhibiting cardiac nerve sprouting and attenuating sympathetic hyper-innervation post-MI and result in reduced inducibility of malignant arrhythmias during EPS in our model, whereas the general myocardial overexpression of Sema3a in the non-MI transgenic mice model ‘resulted in’ too much myocardial Sema3a which was linked with the observed catecholamine supersensitivity and prolongation of the action potential duration. Further experiments on myocardial Sema3a overexpression in mice MI model are essential to clarify this hypothesis.

Both ventricular arrhythmias and left ventricular dysfunction are related to mortality risk 40. Previous study show impaired LVEF is a predictor of the presence of ventricular arrhythmias, but the clear relationship between ventricular arrhythmias and left ventricular dysfunction after myocardial infarction is a controversial issue 1. Our data show no significance among LVEF, LVDs and LVDd of MLV, SLV and PBS group. Thus, the impact of haemodynamic parameters on arrhythmias might be minimal on the observed differences on arrhythmias score and inducibility of VT.

Caution is needed to explore this experimental study results to humans and the impact of increased myocardial Sema3a activity (expression) in MI patients, which could be quite different as we observed in this rat MI model. Myocardial Sema3a overexpression in large animal model might give more hints on potential effects of myocardial Sema3a expression in humans. Nevertheless, our study clearly shows that modulating myocardial Sema3a might be a potential therapeutic option for reducing post-MI malignant arrhythmias and SCD.

## Study limitation

It should be mentioned that a chronic study observing the impact of myocardial overexpression of Sema3a on long-term survival, left ventricular remodelling and telemetry monitored ‘natural’ arrhythmias in this model is essential to conclude the ‘general’ effect of myocardial overexpression of Sema3a in this model. Moreover, a study exploring the role of down-regulating/inhibiting myocardial Sema3a could highlight the importance of myocardial Sema3a balance post-MI in this model.

## Conclusions

Our data show excessive myocardial reinnervation after infarction in this model. This process could be modulated by myocardial overexpression of Sema3a. Myocardial overexpression of Sema3a after infarction can reduce the inducibility of ventricular arrhythmias as a result of attenuated sympathetic reinnervation. This may offer a new potential clinical therapeutic approach for reducing ventricular arrhythmias and SCD post-myocardial infarction.
